# Supplementary material for: ‘Your Tube’: the role of different diets in children who are gastrostomy fed: protocol for a mixed methods exploratory sequential study
Source: BMJ Open. 2019 Oct 9;9(10):e033831. doi: 10.1136/bmjopen-2019-033831 (PMC6797291; doi:10.1136/bmjopen-2019-033831)
Supplement: Author's manuscript [file bmjopen-2019-033831.draft_revisions.pdf]

BMJ Open is committed to open peer review. As part of this commitment we make the peer review history of every article we publish publicly available.

When an article is published we post the peer reviewers' comments and the authors' responses online. We also post the versions of the paper that were used during peer review. These are the versions that the peer review comments apply to.

The versions of the paper that follow are the versions that were submitted during the peer review process. They are not the versions of record or the final published versions. They should not be cited or distributed as the published version of this manuscript.

BMJ Open is an open access journal and the full, final, typeset and author-corrected version of record of the manuscript is available on our site with no access controls, subscription charges or pay-per-view fees (http://bmjopen.bmj.com).

If you have any questions on BMJ Open's open peer review process please email info.bmjopen@bmj.com

BMJ Open

'YourTube' The Role of Different Diets in Children who are Gastrostomy Fed; a mixed methods exploratory sequential study

Journal:	*BMJ Open*
Manuscript ID	bmjopen-2019-033831
Article Type:	Protocol
Date Submitted by the Author:	23-Aug-2019

Complete List of Authors:	Taylor, Johanna; Mental Health and Addiction Research Group, Department of Health Sciences, University of York<br>O'Neill, Mark; University of York<br>Maddison, Jane; University of York,<br>Richardson, Gerry; University of York, Centre for Health Economics<br>Hewitt, Catherine; The University of York, Health Sciences<br>Horridge, Karen; South Tyneside and Sunderland NHS Foundation Trust<br>Cade, Janet; University of Leeds<br>McCarter, Alison; Somerset Partnership NHS and Social Care Trust<br>Beresford, Bryony; University of York, Social Policy Research Unit<br>Fraser, Lorna; University of York,

Keywords:	gastrostomy, home-blended, Community child health < PAEDIATRICS

SCHOLARONE™
Manuscripts

'YourTube' The Role of Different Diets in Children who are Gastrostomy Fed; a mixed methods exploratory sequential study

Taylor J[1,3], O'Neill M[1,3], Maddison J[2,3], Richardson G[4], Hewitt CE[1], Horridge K[5], Cade JE[6], McCarter A[7], Beresford B[2,3], Fraser LK[1,3]

[1] Department of Health Sciences, University of York, UK

[2] Social Policy Research Unit, University of York, UK

[3] Martin House Research Centre, University of York, UK

[4] Centre for Health Economics, University of York, UK

[5] South Tyneside and Sunderland NHS Foundation Trust, UK

[6] Nutritional Epidemiology Group, School of Food Science and Nutrition, University of Leeds, UK

[7] Somerset Partnership NHS Trust, UK

Corresponding Author:

Dr Lorna Fraser

Department of Health Sciences

Area 2 Seebohm Rowntree Building

Heslington

YORK

YO10 5DD

lorna.fraser@york.ac.uk

01904 321889

Word count: 3916

Tables: 2

Figures: 0

For peer review only - http://bmjopen.bmj.com/site/about/guidelines.xhtml

Abstract

Introduction

Increasing numbers of children require having all, or part, of their nutritional intake via gastrostomy. More parents are using home-blended meals to feed their children, with many reporting beneficial effects such as improved gastro-oesphageal reflux, less constipation and less distress in their child.

This study aims to identify the important outcomes of tube feeding in this population, compare the safety, outcomes and resource use of those on a home-blended diets compared to a formula diet, and assess feasibility of long-term follow-up of children recruited to this study.

Methods & Analyses

This mixed methods study of children (age 6 months to 18 years) who are gastrostomy-feed dependent recruited via general, community and specialist paediatric and dietetic services.

Workstream 1: a qualitative study involving semi-structured interviews with parents (n~20) and young people (n~5-10), and focus groups with health professionals (n~25) will provide evidence of appropriate outcome measures and the feasibility/acceptability of proposed data collection methods for WS2. It will gather data on: desired outcomes of gastrostomy feeding, variability in diets and reasons; use of oral feeding; perceived benefits of the alternative diets, resources associated with gastrostomy feeding, and safety issues. Data will be analysed using thematic analysis.

Workstream 2*:* a cohort study of 300 children who are gastrostomy fed. Data will be collected at months 0, 9 and 18 from parents, children (if appropriate) and clinicians using standardised measures and questionnaires developed specifically for the study. Data collected will include gastrointestinal symptoms, health and other outcomes (child, parent), dietary intake, anthropometry, healthcare usage, safety outcomes and resource use. Outcomes in the home-blended and formula groups will be compared using appropriate multiple regression analyses.

Ethics and dissemination

The study has been approved by a research ethics committee (REC reference:19/YH/0028).

Results will be disseminated through publications and presentations for professionals and families.

Registration details

ISRCTN13977361

For peer review only - http://bmjopen.bmj.com/site/about/guidelines.xhtml

Strengths and limitations

- A multi-centre study of a large number of children who are gastrostomy fed
- Key outcomes will be determined from qualitative work with parents, young people and professionals
- The study will use validated measures for gastrointestinal symptoms, nutritional intake, health and other outcomes, and quality of life data collection (PedsQL)
- This is an observational study with no randomisation therefore confounding and bias may be of concern.

Introduction

There are growing numbers of children with complex health conditions who are dependent upon medical technologies to maintain their health, and gastrostomy (or enteral) feeding is one such technology. The authors' own analyses of inpatient hospital (Hospital Episodes Statistics) data found that among children with life-limiting conditions (LLC)[1] in England, the number having permanent gastrostomy surgery each year has risen from 183 in 2000/01 to 1004 in 2014/15. In 2014/2015, the total number of children, with a LLC, aged 0-19 in England who have ever had a gastrostomy was 10,154. This is much higher than published estimates of ~430 children [2].

Children requiring some or all of their nutrition via gastrostomy tubes have a wide range of underlying diagnoses including neurodisability, inherited metabolic diseases, congenital cardiac conditions, cystic fibrosis, gastrointestinal diseases and cancer.

At present, in the UK, the recommended feed for children on enteral feeding is commercially produced complete liquid nutrition (formula), prescribed by the child's dietitian [3]. However there is a growing body of parents who are interested in and/or choosing to feed their children meals they have prepared themselves which are then liquidised so they can be administered via a gastrostomy (referred to forthwith as 'home-blended foods') [4-6]. Parents choosing to use home-blended foods have reported benefits such as improved gastro-oesophageal reflux, less constipation and less distress in their child [7]. There are also perceived psychosocial benefits: it may fulfil parents' need to nurture, and the child is not excluded from sharing the same food as the rest of the family. Prescribed formula, in contrast, is regarded as a medical product rather than food[6].

Limited research evidence [8] and reports from clinicians suggest that the long-term use of gastrostomy feeds for children with complex health conditions can result in complications including progressive feed intolerance/ gut failure. [8]. There are suggestions that a home-blended diet may reduce the risk of gut failure but there is currently no evidence to support this.

Recent national surveys of paediatric dietitians in the UK [4] and the US [5] both found that more than half of respondents would recommend the use of a home-blended diet (56 and 58% respectively). In the UK, however, that recommendation was to use home-blended food as a supplement to formula feeds rather than their exclusive use.

At the same time, concerns have been raised by professional organisations, including the European Society for Paediatric Gastroenterology Hepatology and Nutrition (ESPGHAN) and British Dietetic Association (BDA), about the risks associated with a diet of home-blended foods. These include: nutritional inadequacy, microbial contamination and blockage of the gastrostomy tube. Policy/position statements from such organisations do not recommend that children (or adults) are

For peer review only - http://bmjopen.bmj.com/site/about/guidelines.xhtml

fed home-blended foods through their gastrostomy tubes. [9] Importantly, it was acknowledged in these guidelines that the evidence for this statement is low and further research is likely to have an impact on this recommendation.

Methods and Analyses

The research question is:

What are the risks, benefits and resource implications for using home-blended food for children with gastrostomy tubes compared to currently recommended formula feeds?

The objectives are:

1. To identify the important outcomes of gastrostomy feeding for parents, young people and health professionals.

2. To assess the safety of home-blended diets for children who are gastrostomy fed compared to liquid formula diets.

3. To identify and quantify the benefits of home-blended diets compared to liquid formula feeds for children who are gastrostomy fed and their parents.

4. To identify and quantify the resources (family and statutory services) required to support home-blended diets compared to formula feeds.

5. To assess whether long-term follow-up of children who are gastrostomy fed is feasible using routine data sources.

This study will use a mixed methods exploratory sequential design [10] with two workstreams (WS), with findings from workstream one informing the design and methods for workstream two.

Workstream 1 (WS1)

Design

Qualitative research with purposefully sampled groups of young people, parents and healthcare professionals will investigate views on a number of topics relevant to informing final decisions regarding the design of the cohort study in workstream 2 (WS2) as well as generating 'stand-alone' evidence on young people's, parents' and professionals' views about gastrostomy feeding and the use of home-blended diets.

Eligibility Criteria

- Parents (n=20) of children and young people (aged from 6 months to 18 years inclusive) who are fed via a gastrostomy.
- Young people (n=5-10) aged 12-18 years currently using a gastrostomy and with no significant cognitive impairments.
- Health professionals who provide or support the nutritional care of children with a gastrostomy, specifically paediatricians (n=6-8), dietitians (n=6-8), children's community nurses (n=6-8), and Speech and Language Therapists (n=6-8).

Recruitment

For peer review only - http://bmjopen.bmj.com/site/about/guidelines.xhtml

We will recruit via general, community and/or specialist paediatric services in English National Health Service (NHS) Trusts and children's nutrition and dietetic services attached to, or working into, these services Services will be selected to represent the broad range of stances regarding home-blended diets (supportive, neutral and unsupportive). Based on estimated numbers of children using a home-blended diet we plan to recruit from 6 NHS Trusts in WS1.

Parents will be sampled based on characteristics such as: child's diet, child's age, primary diagnosis, complexity (i.e. number of co-morbidities) and feeding history.

Healthcare professionals will be sampled to ensure representation of: the range of clinicians who support children who are gastrostomy fed, stance on home-blended diets, and experience of supporting children using home-blended diets.

For parents and young people, research staff/clinicians will identify eligible participants and provide the study team with an anonymised list, detailing sampling characteristics. The study team will select which participants to approach based on the purposive sampling criteria. The site research staff/clinicians will then approach the selected participants in clinic or via post. Parents and young poeple who return response forms will be contacted by the study team and an interview time and date will be arranged.

Data Collection

Individual interviews (young people, parents) and focus groups (professionals) will be used to collect data. Parents will be offered the choice of telephone or face-to-face interview. Consent will be recorded at the start of the interview/focus group. For young people aged 12- 15 years, child assent and parent consent will be obtained.

Interview/focus group schedules will cover the following topics (tailored to the characteristics of the interviewee(s)):

- typical diet followed and factors which may affect adherence to that diet
- in terms of blended diets, factors influencing decision to use diet, types of food comprising diet, parental management of diet, support and guidance offered and adherence to guidance
- desired and observed immediate and longer-term health and quality of life outcomes (including unanticipated and/or undesirable) for the child of gastrostomy feeding and perceived impacts of the type of diet used;
- observed symptoms associated with gastrostomy feeding (e.g. reflux, constipation) and impacts of type of diet on symptoms;
- perceived outcomes for parents of their child being fed by gastrostomy, and impacts of type of diet on these outcomes;
- perceived/experienced risks/safety issues and other drawbacks associated with gastrostomy feeding, including the type of diet used;
- reported/perceived costs to families and the NHS (financial, time) of using gastrostomies and the impact of type of diet on those costs.

In addition, interviews with parents will explore views regarding feasibility and acceptability (in terms of parent participation) of the proposed design of the cohort study (e.g. proposed recruitment methods, collecting nutritional data, respondent burden and retention strategies).

For peer review only - http://bmjopen.bmj.com/site/about/guidelines.xhtml

For interviews with young people who have communication impairments, we will use their preferred communication systems and, if necessary, use or create visual tools (for example Talking Mats^{TM}[11]) to facilitate the interview [12].

With participants' permission, interviews/focus groups will be audio-recorded and verbatim transcripts obtained.

Data Analysis

We will use thematic analysis techniques [13] to analyse the data to identify and describe experiences of gastrostomy feeding, ways in which blended-food diets are being implemented, outcomes that are important across the sample, resource implications and complications associated with blended feeds, and to examine the acceptability and appropriateness of piloted measurement tools. Specifically, we will use the Framework approach [14] to facilitate systematic data management and ensure audit trails of the data management process:

1. Researchers familiarise themselves with the data, and identify themes and key issues.
2. Based on identified themes and any a priori issues (e.g. acceptability of proposed WS2 data collection tools, outcomes associated with gastrostomy feeding, resource use), an index of themes is constructed (the thematic framework).
3. Data are then indexed according to which theme(s) in the analytical framework they relate to. The indexed data from each case (e.g. participant, focus group) are summarised onto a series of thematic matrices (or charts). Each chart is divided into columns, allowing relevant data to be organised according to sub-themes/issues. A single row on each chart holds one participant's data. Thus reading along a row provides an overview of everything an individual spoke about in terms of a specific issue. Reading down the chart (or down a column) allows comparison between participants'.
4. The final stage of analysis involves 'reading' of the charts, composing 'analytical notes' which describe the data and developing interpretation and hypotheses which are then tested against the charts and raw data. To start, data will be analysed by participant group after which there will be a process of comparison between groups.

For peer review only

Integrating WS1 findings into final decision-making regarding WS2

WS1 findings will be presented to an expert study steering committee (SSC) comprising parent, clinical and academic expertise. The SSC will be tasked, in discussion with the research team, with agreeing which outcomes to measure in WS2 and selecting appropriate measurement / data collection tools for these in terms of feasibility (e.g. respondent burden) and comprehensiveness. Where additional outcome domains not included in the original protocol are identified in WS1, candidate outcome measures will be identified by the research team and presented to the SSC. The SSC will also review WS1 findings regarding the need to include further descriptive and predictor variables for WS2, and the team's proposed means of collecting data on these.

<u>Workstream 2</u>

Design

A prospective cohort study with an initial 18 month follow-up period but also including an assessment of the potential for long term (10 years+) follow-up using routine data sources to measure key outcomes for these children.

Eligibility criteria

Children (aged 6 months to 18 years inclusive) who receive most of their nutrition via gastrostomy tube. Parents of participating children will also take part in the study. We are including child and parent dyads in order to measure child and parent outcomes (e.g. quality of life); therefore, children who do not live with a biological or adoptive parent are not eligible to take part. The study is limited to families who reside in England (see Table 1 for summary of inclusion and exclusion criteria).

Table 1Eligibility Criteria

	ELIGIBLE	***INELIGIBLE***
A	Child is at least 6 months old and under 19 years	Infants up to 6 months and young people who are 19 years and older
B	Child is gastrostomy feed dependent	Child has another type of feeding tube (e.g. nasogastric, jejunostomy)
C	Child receives most or all of their nutrition via the gastrostomy	
D	Child is living with parent(s): biological or adoptive	Child is not living with a parent (e.g. in residential setting or foster care)
E	Family resident in England	Family not resident in England

Sample Size

Given that no data are available on the exact proportion of children who receive formula feeds versus a home-blended diet in England, various scenarios were explored to ensure that the study would be adequately powered. A sample size of 300 for the analysis (assuming there are twice as many formula fed as home blended) should enable us to estimate proportions within each group to within a margin of error of ≤10% and continuous measures (e.g. Pediatric Quality of Life Inventory(PEDSQL)assuming standard deviation 20) to within a standard error of 4 points.

Recruitment

As per WS1, children and their parents will be recruited via general, community and/or specialist paediatric services in English NHS Trusts, and children's nutrition and dietetic services attached to, or working into, these services. Based on estimated numbers of children using a home-blended diet we plan to recruit from around 20 NHS Trusts. For WS2 we will also plan to recruit from children's hospices in England and via social media, as recommended by the parent advisors for the study.

Families will be recruited primarily in routine clinic appointments with paediatricians or dietitians, but families may also be invited by post, telephone, and via social media. In WS2, families must be supported by a recruiting NHS Trust or children's hospice to take part, in order for clinical data to be

For peer review only - http://bmjopen.bmj.com/site/about/guidelines.xhtml

obtained in the study. In WS2, clinicians will introduce the study and obtain consent from parents and children where possible for the study team to contact them. Consent to participate in the study will be sought by the study team, with appropriate consent / assent processes used depending on the age and capacity of participating children. For young adults (16-18yrs), an assessment of capacity in line with the Mental Capacity Act, will be undertaken by the clinician at the consent to contact stage.

All families will receive a full study information pack about the study, which will contain relevant participant information sheets and study consent forms depending on the child / young person's capacity. For young adults who lack capacity, we will identify an appropriate personal consultee to provide advice about the young adult's views and wishes about taking part in the study, using appropriate consultee information sheets and consultee declaration forms.

Consent processes are as follows:

- *Young adults age 16-18 years with capacity:* young adults and parents will provide separate consent for their own participation.
- *Young adults age 16-18 years without capacity:* no consent will be taken. Young adults will take part in the study if the consultee advises that they would not have any objections to taking part. Parents will consent separately for their own participation in the study.
- *Children and young people age 7-15 years who can understand information about the study and express an opinion about taking part:* parents will provide consent and the child / young person will provide written or verbal assent. A simplified version of the assent form will be used for children aged 7-11 years, with a standard version used for young people aged 12-15 years). Parents will provide separate consent for their own participation.
- *All other children and young people under the age of 16 years:* parents will consent for their child and themselves.

Data Collection

Data will be collected at three time points: at baseline and then at 9 and 18 months. At each time point, data on a range of outcomes as well as relevant clinical and feeding information will be collected from parents/children/young people and clinicians See Table 2 for summary of proposed outcomes and data sources (these are subject to change following WS1).

The majority of the data will be collected via parent questionnaire administered according to parent preference (postal vs on-line; parents will also be offered telephone interview). Parents whose children will also be required to provide dietary information via the online myfood 24 tool,[15] or via a paper food diary or telephone call with the research team if using the online tool is not possible. Where appropriate, participating children and young people will also be asked to complete a short questionnaire (e.g. to self-report quality of life). Up to three reminders via text and/or post will be used at each time point. A small incentive voucher of £20 will be provided to each family after return of the questionnaires at each time point.

Clinical information (e.g. diagnoses, medications, anthropometry) will be collected from children's paediatrician and/or dietitian. Dietitians will also provide details about formula feeds used by participating children. Finally, we will use routine healthcare data through linkage undertaken by NHS Digital to the Hospital Episodes Statistics (HES) data (inpatient, A&E, outpatient) and the Office for National Statistics (ONS) death certificate data.

For peer review only - http://bmjopen.bmj.com/site/about/guidelines.xhtml

Table 2: Proposed variables and data sources for WS2

Variable	Type of Variable	Proposed Measure	Source	Timings of data collection (months)		
				0	9	18
**Participant characteristics / predictors**						
Age	Predictor	Date of birth	Parent	√		
Ethnicity		Census groups	Parent	√		
Deprivation		Index of Multiple Deprivation (based on postcode)	Parent	√	√	√
Parental educational attainment		Census groups	Parent	√		
Household composition		Number of children, marital / living status	Parent	√	√	√
Diagnosis			Paediatrician	√		
Co-morbidities			Paediatrician	√	√	√
All Medications			Paediatrician	√	√	√
Complexity		Disability Complexity Scale[16 17]	Paediatrician	√	√	√
Length of time gastrostomy fed at T0		Months/years	Parent	√		
**Comparator**						
Diet: Formula/blended	Main grouping variable		Parent	√	√	√
**Outcomes**						
Nutritional content of feeds	Outcome	Commercial for formula	Dietitian/Parent	√	√	√
		myfood24[15]	Parent	√	√	√
Anthropometric data	Outcome	Height or length<br>Weight	Dietician<br>Paediatrician	√	√	√

For peer review only - http://bmjopen.bmj.com/site/about/guidelines.xhtml

		Triceps skinfold thickness or mid arm circumference				
Gastrointestinal symptoms	Outcome	PedsQL Gastrointestinal[18]	Parent/ child	√	√	√
Child Quality of Life	Outcome	PedsQL generic module[19]	Parent/ child	√	√	√
Parental Quality of Life	Outcome	EQ5D5L[20]<br>Parenting Morale Index[21]	Parent	√	√	√
Healthcare use	Outcome	Client service receipt inventory[22]	Parent	√	√	√
	Outcome	No. hospital admissions<br>No. Accident and Emergency (A and E) attendances	HES data	√	√	√
Safety	Outcome	Tube blockages<br>No. of infections stoma<br>No. of gastrointestinal infections	Parent			
	Outcome	Number of hospital admissions and A and E attendance associated with child's gastrostomy/diet	HES & Parent	√	√	√
Family resource use	Outcome	Time preparing feeds; impact on other caring/parenting<br>Financial costs	Parent	√	√	√
Non-staff NHS resource use	Outcome	Cost of formula and packaging<br>Dietetic resources	Dietitian	√	√	√

Data Analyses

The data quality of each data item collected will be assessed when the data is collected or received by the research team. Appropriate attempts will be made to obtain missing or out of value data. A review of the collected data will be undertaken after the first 25 participants have completed baseline questionnaires to check for any systematic issues with the data collection.

A statistical analysis plan will be developed and signed off prior to analysis. Analysis will follow STROBE [23] and RECORD [24] guidelines. Descriptive statistics of clinical and demographic characteristics of the study population at baseline will be used to examine differences between the groups of children who are predominantly formula fed and those who use home-blended diets.

For peer review only - http://bmjopen.bmj.com/site/about/guidelines.xhtml

Children will be grouped into those who are on a predominantly blended diet or formula diet at baseline by:

- Home-blended group if most of their nutritional intake is provided via home blended diet. This categorisation will be informed by WS1 and in consultation with the SSC.
- Formula fed if most of their diet comes from formula.

Most of the proposed outcome measures will require scoring or aggregation before the statistical modelling can be undertaken:

- The Peds QL generic scale[19] and PedsQL Gastrointestinal symptoms module[18] will be scored as per the guidelines and transformed to a score of 0-100.
- The height (or length) and weight will be used to calculate an age and sex adjusted body mass index (bmi sds).
- The myfood24 data programme analyses the nutritional content the home-blended diet and will compute the calorie intake and the macro and micro nutrient content of the feed and any oral feeds. The same data for the formula fed group will have been obtained, via the dietitian, from the commercial supplier.
- The parent reported number of site infections and other tube related complications will reported as total counts for each child.
- The diagnostic (ICD10[25]) and procedural codes (OPCS) in the HES data will be used to identify admissions which were related to complications of the gastrostomy tubes or infections. The number of admissions and A & E attendances will be calculated for each child. Length of stay for each admission will also be calculated for the resource use analyses.
- Parent quality of life: the EQ5D VAS is scored 0-100 and the 5 component scale of the EQ5D-5L will be converted to a single score using a UK specific value set [26] . The 10 item Parenting Morale Index is scored from 0-100.

For all outcomes we will report the baseline score, follow-up scores and change score.

Assessing safety (objective 2) and benefits (objective 3):

The safety and benefits of blended diet compared to formula diet will be assessed using multivariable regression analyses. The type of regression will depend on the outcome of interest; logistic (tube blockage, appropriate nutritional content; yes/no), linear (PEDSQL gastrointestinal module score, BMI sds or upper arm circumference, calories, PMI, EQ5D), Poisson or negative binomial (number of A & E or hospital admissions for infections or complications of gastrostomy tube). Each analysis will account for the multiple confounding factors in this population (age, underlying diagnoses, comorbidities, outpatient attendance, parental factors, socio-economic status) and the main covariate of interest will be feeding status (blended vs formula). Study site will be added as a random effect to the models to allow for site level variation. Estimates and 95% CIs will be reported from the regression model for each outcome measure.

The flow of participants through the study will be detailed including the number of individuals contributing to each analysis. The amount of missing data will be summarised for each outcome measure and multiple imputation will be used to assess the robustness of the results. Results will be compared to the complete case analyses and important differences discussed. Sensitivity analyses will be considered to explore departures from the MAR assumption.

For peer review only - http://bmjopen.bmj.com/site/about/guidelines.xhtml

Measurement of cost and outcomes (objectives 3 and 4)

There is a lack of robust evidence around the cost-effectiveness of alternative feeds for gastrostomy fed children. To address this, we will describe the costs and outcomes for those children with a formula diet and with a –blended diet(addressing objectives 3 and 4). The formula feed group will act as the treatment as usual.

Generating cost estimates

Unit costs for healthcare interactions will be collected from published sources (for example, PSSRU Unit Costs of Health and Social Care) and applied to the relevant resource use. The costs of healthcare interactions will be calculated by the product of unit cost and resource use analyses.

The cost of non-healthcare interactions, including the cost of the blended diet constituents and time taken to prepare will be estimated separately using published estimates where feasible. Sensitivity analyses will be conducted where alternative assumptions would generate substantially different cost estimates (for example, if there are substantial differences between parental report and HES data).

Generating estimates of outcomes

We will describe and summarise estimates of parent changes in HRQoL (EQ5D-5L) and child (either PEDS QL or QoL questionnaire [27]). We will describe these for both groups within the cohort.

We will report total costs, mortality and adverse event rates associated with both home-blended and formula feeds in a cost-consequences framework.

Long term follow up (objective 5)

The utility of routine data sources as an option for long term follow up of study participants will be assessed by examining the concordance between parent reported data on A and E visits and hospital admissions due to infections or complications of their gastrostomy tube with HES data for the corresponding time period. Concordance will be assessed using the kappa statistic both for the total sample and separately by the home-blended and formula fed groups.

If there is concordance between the parental reported infection and gastrostomy related healthcare usage and HES data, long term follow-up would be possible by obtaining further extracts of HES data and ONS death certificate data. The HES data will provide future information on admissions and A&E visits due to infections and complications of the gastrostomy (blockages, revisions, replacements). The ONS data will provide date and cause(s) of death if the child has died.

Patient and Public Involvement

Six parents whose children were gastrostomy fed were involved in the development of the study design and methods, and plans for public involvement. In particular, they helped to identify outcomes to propose for WS2, develop appropriate recruitment methods for both workstreams (e.g. recommended that we use social media), and provided advice about how to name the study (i.e. using the term different diets rather than blended diets).

During the study, our primary mechanism for public involvement will be via a Project Advisory Panel comprising 4-5 parents and 2-3 young people with gastrostomy experience. The panel will meet twice per year at key points in the study, and be involved at other times when needed via telephone, email

For peer review only - http://bmjopen.bmj.com/site/about/guidelines.xhtml

or in person depending on the task. Panel members will be supported by the study team, and receive training for specific tasks when needed.

Members of the panel will be involved in the following:

Study oversight: A minimum of two members of the panel will also be members of the SSC.

Developing study materials: reviewing participant information sheets, consent forms and interview schedules; piloting of WS1 topic guides and materials; development of guidance for parents taking part in WS2; piloting of WS2 questionnaires and data collection tools.

WS2 study design: selection of outcomes to be included in WS2, and input on the acceptability and feasibility of proposed measurement tools.

Study interpretation: input on the meaning of study findings, and development of key messages for policy and practice

Study dissemination: help to produce information resources for parents, children and young people at the end of the study; identify routes for dissemination and assist in the dissemination of the study outputs directly via their own networks.

Ethics and dissemination

Approvals from the English National Health Service Research Ethics Committee and Health Research Authority have been obtained for this study (REF 19/YH/0028).

The study findings will be disseminated in a final report to the funder, academic publications and resources for professionals and parents, children and young people.

References

1. Together for Short Lives. A Guide to Children's Palliative Care. 4th ed. Bristol, 2018.
2. Smith TM, Hirst A, Stratton R. Artificial Nutition Support in the UK 2000-2010. Annual BANS Report: BANS 2011.
3. British Dietetic Association. Practice Toolkit; Liquidised Food via Gastrostomy Tube 2017 [Available from: https://www.bda.uk.com/professional/practice/liquidisedtoolkit accessed 1/9/2017 2017.
4. Armstrong J, Buchanan E, Duncan H, et al. Dietitians' perceptions and experience of blenderised feeds for paediatric tube-feeding. *Archives of Disease in Childhood* 2017;102(2):152-+. doi: 10.1136/archdischild-2016-310971
5. Johnson TW, Spurlock A, Pierce L. Survey Study Assessing Attitudes and Experiences of Pediatric Registered Dietitians Regarding Blended Food by Gastrostomy Tube Feeding. *Nutrition in Clinical Practice* 2015;30(3):402-05. doi: 10.1177/0884533614564996
6. Breaks A, Smith C, Bloch S, et al. Blended diets for gastrostomy fed children and young people: a scoping review. *Journal of Human Nutrition and Dietetics* 2018;31(5):634-46. doi: 10.1111/jhn.12563
7. Brown S. Blended food for enteral feeding via a gastrostomy. *Nursing Children and Young People* 2014;26(9):16-20.
8. Siden H, Tucker T, Derman S, et al. Pediatric Enteral Feeding Intolerance: A new prognosticator for children with life-limiting illness? *Journal of Palliative Care* 2009;25(3):213-17.
9. Romano C, van Wynckel M, Hulst J, et al. European Society for Paediatric Gastroenterology, Hepatology and Nutrition Guidelines for the Evaluation and Treatment of Gastrointestinal and Nutritional Complications in Children With Neurological Impairment. *Journal of Pediatric Gastroenterology and Nutrition* 2017;65(2):242-64. doi: 10.1097/mpg.0000000000001646
10. Creswell JW, Plano Clark V, Hanson W. Handbook of mixed methods in social and behavioral research. *Advanced Mixed Methods Reserach Designs* 2003;212

For peer review only - http://bmjopen.bmj.com/site/about/guidelines.xhtml

11. University of Stirling. Talking Mats 2018 [Available from: https://www.talkingmats.com/ accessed 15/3/2018 2018.
12. Rabiee P, Sloper P, Beresford B. Doing research with children and young people who do not use speech for communication. *Children & Society* 2005;19(5):385-96. doi: 10.1002/chi.841
13. Miles M, Huberman M, J S. Qualitative Data Analysis. London: SAGE 2013.
14. Ritchie J, J L. Qualitative Research Practice. London SAGE 2003.
15. Carter MC, Hancock N, Albar SA, et al. Development of a New Branded UK Food Composition Database for an Online Dietary Assessment Tool. *Nutrients* 2016;8(8) doi: 10.3390/nu8080480
16. Horridge KA, Harvey C, McGarry K, et al. Quantifying multifaceted needs captured at the point of care. Development of a Disabilities Terminology Set and Disabilities Complexity Scale. *Developmental Medicine and Child Neurology* 2016;58(6):570-80. doi: 10.1111/dmcn.13102
17. Horridge KA, McGarry K, Williams J, et al. Prospective pilots of routine data capture by paediatricians in clinics and validation of the Disabilities Complexity Scale. *Developmental Medicine and Child Neurology* 2016;58(6):581-88. doi: 10.1111/dmcn.13101
18. Varni JW, Bendo CB, Shulman RJ, et al. Interpretability of the PedsQL (TM) Gastrointestinal Symptoms Scales and Gastrointestinal Worry Scales in Pediatric Patients With Functional and Organic Gastrointestinal Diseases. *Journal of Pediatric Psychology* 2015;40(6):591-601. doi: 10.1093/jpepsy/jsv005
19. varni JW. The PedsQL measurement model for the pediatric quality of life inventory 2017 [Available from: http://www.pedsql.org/.
20. Herdman M, Gudex C, Lloyd A, et al. Development and preliminary testing of the new five-level version of EQ-5D (EQ-5D-5L). *Quality of Life Research* 2011;20(10):1727-36. doi: 10.1007/s11136-011-9903-x
21. Benzies KM, Trute B, Worthington C, et al. Assessing Psychological Well-Being in Mothers of Children with Disability: Evaluation of the Parenting Morale Index and Family Impact of Childhood Disability Scale. *Journal of Pediatric Psychology* 2011;36(5):506-16. doi: 10.1093/jpepsy/jsq081
22. Chisholm D, Knapp MRJ, Knudsen HC, et al. Client Socio-Demographic and Service Receipt Inventory - European Version: Development of an instrument for international research - EPSILON Study 5. *British Journal of Psychiatry* 2000;177:S28-S33. doi: 10.1192/bjp.177.39.s28
23. von Elm E, Altman DG, Egger M, et al. The Strengthening the Reporting of Observational Studies in Epidemiology (STROBE) Statement: Guidelines for Reporting Observational Studies. *PLOS Medicine* 2007;4(10):e296. doi: 10.1371/journal.pmed.0040296
24. Benchimol EI, Smeeth L, Guttmann A, et al. The REporting of studies Conducted using Observational Routinely-collected health Data (RECORD) Statement. *PLOS Medicine* 2015;12(10):e1001885. doi: 10.1371/journal.pmed.1001885
25. World Health Organisation. International Statistical Classification of Diseases and Related Health Problems. 10 ed. Geneva, Switzerland: World Health Organisation 1992.
26. Devlin NJ, Shah KK, Feng Y, et al. Valuing health-related quality of life: An EQ-5D-5L value set for England. *Health Economics* 2018;27(1):7-22. doi: 10.1002/hec.3564
27. Zaidi T, Sudall C, Kauffmann L, et al. Physical outcome and quality of life after total esophagogastric dissociation in children with severe neurodisability and gastroesophageal reflux, from the caregiver's perspective. *Journal of Pediatric Surgery* 2010;45(9):1772-76. doi: 10.1016/j.jpedsurg.2010.04.022

Author's contributions

MO and LF drafted the first version of the manuscript. All authors contributed to the study design and reviewed the manuscript prior to submission.

Funding statement

This study is funded by the National Institute for Health Research Health Technology Assessment programme (ref 17/76/06). This was a commissioned call so the funder identified the topic area for this study but will have no role in the study design, data analyses or conclusions in this study.

For peer review only - http://bmjopen.bmj.com/site/about/guidelines.xhtml

Competing interests statement

None declared

For peer review only

For peer review only - http://bmjopen.bmj.com/site/about/guidelines.xhtml

BMJ Open

‘YourTube’ The Role of Different Diets in Children who are Gastrostomy Fed; protocol for a mixed methods exploratory sequential study

Journal:	*BMJ Open*
Manuscript ID	bmjopen-2019-033831.R1
Article Type:	Protocol
Date Submitted by the Author:	05-Sep-2019
Complete List of Authors:	Taylor, Johanna; Mental Health and Addiction Research Group, Department of Health Sciences, University of York<br>O'Neill, Mark; University of York<br>Maddison, Jane; University of York,<br>Richardson, Gerry; University of York, Centre for Health Economics<br>Hewitt, Catherine; The University of York, Health Sciences<br>Horridge, Karen; South Tyneside and Sunderland NHS Foundation Trust<br>Cade, Janet; University of Leeds<br>McCarter, Alison; Somerset Partnership NHS and Social Care Trust<br>Beresford, Bryony; University of York, Social Policy Research Unit<br>Fraser, Lorna; University of York,
**Primary Subject Heading**:	Paediatrics
Secondary Subject Heading:	Gastroenterology and hepatology
Keywords:	gastrostomy, home-blended, Community child health < PAEDIATRICS

SCHOLARONE™
Manuscripts

'YourTube' The Role of Different Diets in Children who are Gastrostomy Fed; protocol for a mixed methods exploratory sequential study

Taylor J[1,3], O'Neill M[1,3], Maddison J[2,3], Richardson G[4], Hewitt C[1], Horridge K[5], Cade J[6], McCarter A[7], Beresford B[2,3], Fraser LK[1,3]

[1] Department of Health Sciences, University of York, UK

[2] Social Policy Research Unit, University of York, UK

[3]Martin House Research Centre, University of York, UK

[4] Centre for Health Economics, University of York, UK

[5] South Tyneside and Sunderland NHS Foundation Trust, UK

[6] Nutritional Epidemiology Group, School of Food Science and Nutrition, University of Leeds, UK

[7] Somerset Partnership NHS Trust, UK

Corresponding Author:

Dr Lorna Fraser

Department of Health Sciences

Area 2 Seebohm Rowntree Building

Heslington

YORK

YO10 5DD

lorna.fraser@york.ac.uk

01904 321889

Word count: 3916

Tables: 2

Figures: 0

For peer review only

Abstract

Introduction

Increasing numbers of children require having all, or part, of their nutritional intake via gastrostomy. More parents are using home-blended meals to feed their children, with many reporting beneficial effects such as improved gastro-oesphageal reflux, less constipation and less distress in their child.

This study aims to identify the important outcomes of tube feeding in this population, compare the safety, outcomes and resource use of those on a home-blended diets compared to a formula diet, and assess feasibility of long-term follow-up of children recruited to this study.

Methods & Analyses

This mixed methods study of children (age 6 months to 18 years) who are gastrostomy-feed dependent recruited via general, community and specialist paediatric and dietetic services.

Workstream 1: a qualitative study involving semi-structured interviews with parents (n~20) and young people (n~5-10), and focus groups with health professionals (n~25) will provide evidence of appropriate outcome measures and the feasibility/acceptability of proposed data collection methods for WS2. It will gather data on: desired outcomes of gastrostomy feeding, variability in diets and reasons; use of oral feeding; perceived benefits of the alternative diets, resources associated with gastrostomy feeding, and safety issues. Data will be analysed using thematic analysis.

Workstream 2*:* a cohort study of 300 children who are gastrostomy fed. Data will be collected at months 0, 9 and 18 from parents, children (if appropriate) and clinicians using standardised measures and questionnaires developed specifically for the study. Data collected will include gastrointestinal symptoms, health and other outcomes (child, parent), dietary intake, anthropometry, healthcare usage, safety outcomes and resource use. Outcomes in the home-blended and formula groups will be compared using appropriate multiple regression analyses.

Ethics and dissemination

The study has been approved by a research ethics committee (REC reference:19/YH/0028).

Results will be disseminated through publications and presentations for professionals and families.

Registration details

ISRCTN13977361

For peer review only - http://bmjopen.bmj.com/site/about/guidelines.xhtml

Strengths and limitations

- A multi-centre study of a large number of children who are gastrostomy fed
- Key outcomes will be determined from qualitative work with parents, young people and professionals
- The study will use validated measures for gastrointestinal symptoms, nutritional intake, health and other outcomes, and quality of life data collection (PedsQL)
- This is an observational study with no randomisation therefore confounding and bias may be of concern.

Introduction

There are growing numbers of children with complex health conditions who are dependent upon medical technologies to maintain their health, and gastrostomy (or enteral) feeding is one such technology. The authors' own analyses of inpatient hospital (Hospital Episodes Statistics) data found that among children with life-limiting conditions (LLC)[1] in England, the number having permanent gastrostomy surgery each year has risen from 183 in 2000/01 to 1004 in 2014/15. In 2014/2015, the total number of children, with a LLC, aged 0-19 in England who have ever had a gastrostomy was 10,154. This is much higher than published estimates of ~430 children [2].

Children requiring some or all of their nutrition via gastrostomy tubes have a wide range of underlying diagnoses including neurodisability, inherited metabolic diseases, congenital cardiac conditions, cystic fibrosis, gastrointestinal diseases and cancer.

At present, in the UK, the recommended feed for children on enteral feeding is commercially produced complete liquid nutrition (formula), prescribed by the child's dietitian [3]. However there is a growing body of parents who are interested in and/or choosing to feed their children meals they have prepared themselves which are then liquidised so they can be administered via a gastrostomy (referred to forthwith as 'home-blended foods') [4-6]. Parents choosing to use home-blended foods have reported benefits such as improved gastro-oesophageal reflux, less constipation and less distress in their child [7]. There are also perceived psychosocial benefits: it may fulfil parents' need to nurture, and the child is not excluded from sharing the same food as the rest of the family. Prescribed formula, in contrast, is regarded as a medical product rather than food[6].

Limited research evidence [8] and reports from clinicians suggest that the long-term use of gastrostomy feeds for children with complex health conditions can result in complications including progressive feed intolerance/ gut failure. [8]. There are suggestions that a home-blended diet may reduce the risk of gut failure but there is currently no evidence to support this.

Recent national surveys of paediatric dietitians in the UK [4] and the US [5] both found that more than half of respondents would recommend the use of a home-blended diet (56 and 58% respectively). In the UK, however, that recommendation was to use home-blended food as a supplement to formula feeds rather than their exclusive use.

At the same time, concerns have been raised by professional organisations, including the European Society for Paediatric Gastroenterology Hepatology and Nutrition (ESPGHAN) and British Dietetic Association (BDA), about the risks associated with a diet of home-blended foods. These include: nutritional inadequacy, microbial contamination and blockage of the gastrostomy tube. Policy/position statements from such organisations do not recommend that children (or adults) are

For peer review only - http://bmjopen.bmj.com/site/about/guidelines.xhtml

fed home-blended foods through their gastrostomy tubes. [9] Importantly, it was acknowledged in these guidelines that the evidence for this statement is low and further research is likely to have an impact on this recommendation.

Methods and Analyses

The research question is:

What are the risks, benefits and resource implications for using home-blended food for children with gastrostomy tubes compared to currently recommended formula feeds?

The objectives are:

1. To identify the important outcomes of gastrostomy feeding for parents, young people and health professionals.

2. To assess the safety of home-blended diets for children who are gastrostomy fed compared to liquid formula diets.

3. To identify and quantify the benefits of home-blended diets compared to liquid formula feeds for children who are gastrostomy fed and their parents.

4. To identify and quantify the resources (family and statutory services) required to support home-blended diets compared to formula feeds.

5. To assess whether long-term follow-up of children who are gastrostomy fed is feasible using routine data sources.

This study will use a mixed methods exploratory sequential design [10] with two workstreams (WS), with findings from workstream one informing the design and methods for workstream two. The research team comprises clinical and methodological experts including applied social scientists (leading WS1) and a clinical epidemiologist leading WS2.

Workstream 1 (WS1)

Design

Phenomenological qualitative research with young people, parents and healthcare professionals. Interviews (young people, parents) and focus groups (healthcare professionals) will be used investigate views on a number of topics relevant to informing final decisions regarding the design of the cohort study in workstream 2 (WS2) as well as generating 'stand-alone' evidence on young people's, parents' and professionals' views about gastrostomy feeding and the use of home-blended diets.

Eligibility Criteria

- Parents (n=20) of children and young people (aged from 6 months to 18 years inclusive) who are fed via a gastrostomy.
- Young people (n=5-10) aged 12-18 years currently using a gastrostomy and with no significant cognitive impairments.
- Health professionals who provide or support the nutritional care of children with a gastrostomy, specifically paediatricians (n=6-8), dietitians (n=6-8), children's community nurses (n=6-8), and Speech and Language Therapists (n=6-8).

For peer review only - http://bmjopen.bmj.com/site/about/guidelines.xhtml

Sampling

Purposeful sampling will be used. arents will be sampled to ensure representation of different diets (formula vs home-blended vs mixed), dietary history (i.e. unchanged since gastrostomy vs change of diet)range of children's ages, duration of gastrostomy feeding, and broad stance of clinicians overseeing child's care regarding home blended diets (i.e. supportive vs unsupportive). Healthcare professionals will be sampled to ensure representation of: the range of healthcare professions (e.g. paediatricians, dieticians) who support children who are gastrostomy fed, stance on home-blended diets, and experience of supporting children using home-blended diets.

Recruitment

We will recruit via general, community and/or specialist paediatric services in English National Health Service (NHS) Trusts and children's nutrition and dietetic services attached to, or working into, these services Services will be selected to represent the broad range of stances regarding home-blended diets (supportive, neutral and unsupportive). Based on estimated numbers of children using a home-blended diet we plan to recruit from 6 NHS Trusts in WS1.

For parents and young people, research staff/clinicians will identify eligible participants and provide the study team with an anonymised list, detailing sampling characteristics. The study team will select which participants to approach based on sampling criteria (see above). The site research staff/clinicians will then approach the selected participants in clinic or via post. Parents and young poeple who return response forms will be contacted by the study team and an interview time and date will be arranged.

Data Collection

Individual interviews (young people, parents) and focus groups (professionals) will be used to collect data. Parents will be offered the choice of telephone or face-to-face interview. Consent will be recorded at the start of the interview/focus group. For young people aged 12- 15 years, child assent and parent consent will be obtained.

Interview/focus group schedules will cover the following topics (tailored to the characteristics of the interviewee(s)):

- typical diet followed and factors which may affect adherence to that diet
- in terms of blended diets, factors influencing decision to use diet, types of food comprising diet, parental management of diet, support and guidance offered and adherence to guidance
- desired and observed immediate and longer-term health and quality of life outcomes (including unanticipated and/or undesirable) for the child of gastrostomy feeding and perceived impacts of the type of diet used;
- observed symptoms associated with gastrostomy feeding (e.g. reflux, constipation) and impacts of type of diet on symptoms;
- perceived outcomes for parents of their child being fed by gastrostomy, and impacts of type of diet on these outcomes;
- perceived/experienced risks/safety issues and other drawbacks associated with gastrostomy feeding, including the type of diet used;

For peer review only - http://bmjopen.bmj.com/site/about/guidelines.xhtml

- reported/perceived costs to families and the NHS (financial, time) of using gastrostomies and the impact of type of diet on those costs.

In addition, interviews with parents will explore views regarding feasibility and acceptability (in terms of parent participation) of the proposed design of the cohort study (e.g. proposed recruitment methods, collecting nutritional data, respondent burden and retention strategies).

For interviews with young people who have communication impairments, we will use their preferred communication systems and, if necessary, use or create visual tools (for example Talking Mats™[11]) to facilitate the interview [12].

With participants' permission, interviews/focus groups will be audio-recorded and verbatim transcripts obtained.

Data Analysis

An inductive approach to data analysis, using thematic analysis techniques [13] will be used to identify and describe experiences of gastrostomy feeding, ways in which blended-food diets are being implemented, outcomes that are important across the sample, resource implications and complications associated with blended feeds, and to examine the acceptability and appropriateness of piloted measurement tools. Specifically, we will use the Framework approach [14] to facilitate systematic data management and ensure audit trails of the data management process:

1. Researchers familiarise themselves with the data, and identify themes and key issues.
2. Based on identified themes and any a priori issues (e.g. acceptability of proposed WS2 data collection tools, outcomes associated with gastrostomy feeding, resource use), an index of themes is constructed (the thematic framework).
3. Data are then indexed according to which theme(s) in the analytical framework they relate to. The indexed data from each case (e.g. participant, focus group) are summarised onto a series of thematic matrices (or charts). Each chart is divided into columns, allowing relevant data to be organised according to sub-themes/issues. A single row on each chart holds one participant's data. Thus reading along a row provides an overview of everything an individual spoke about in terms of a specific issue. Reading down the chart (or down a column) allows comparison between participants'.
4. The final stage of analysis involves 'reading' of the charts, composing 'analytical notes' which describe the data and developing interpretation and hypotheses which are then tested against the charts and raw data. To start, data will be analysed by participant group after which there will be a process of comparison between groups.

Integrating WS1 findings into final decision-making regarding WS2

WS1 findings will be presented to an expert study steering committee (SSC) comprising parent, clinical and academic expertise. The SSC will be tasked, in discussion with the research team, with agreeing which outcomes to measure in WS2 and selecting appropriate measurement / data collection tools for these in terms of feasibility (e.g. respondent burden) and comprehensiveness. Where additional outcome domains not included in the original protocol are identified in WS1, candidate outcome

For peer review only - http://bmjopen.bmj.com/site/about/guidelines.xhtml

measures will be identified by the research team and presented to the SSC. The SSC will also review WS1 findings regarding the need to include further descriptive and predictor variables for WS2, and the team's proposed means of collecting data on these.

Workstream 2

Design

A prospective cohort study with an initial 18 month follow-up period but also including an assessment of the potential for long term (10 years+) follow-up using routine data sources to measure key outcomes for these children.

Eligibility criteria

Children (aged 6 months to 18 years inclusive) who receive most of their nutrition via gastrostomy tube. Parents of participating children will also take part in the study. We are including child and parent dyads in order to measure child and parent outcomes (e.g. quality of life); therefore, children who do not live with a biological or adoptive parent are not eligible to take part. The study is limited to families who reside in England (see Table 1 for summary of inclusion and exclusion criteria).

Table 1Eligibility Criteria

	ELIGIBLE	***INELIGIBLE***
A	Child is at least 6 months old and under 19 years	Infants up to 6 months and young people who are 19 years and older
B	Child is gastrostomy feed dependent	Child has another type of feeding tube (e.g. nasogastric, jejunostomy)
C	Child receives most or all of their nutrition via the gastrostomy	
D	Child is living with parent(s): biological or adoptive	Child is not living with a parent (e.g. in residential setting or foster care)
E	Family resident in England	Family not resident in England

Sample Size

Given that no data are available on the exact proportion of children who receive formula feeds versus a home-blended diet in England, various scenarios were explored to ensure that the study would be adequately powered. A sample size of 300 for the analysis (assuming there are twice as many formula fed as home blended) should enable us to estimate proportions within each group to within a margin of error of ≤10% and continuous measures (e.g. Pediatric Quality of Life Inventory(PEDSQL)assuming standard deviation 20) to within a standard error of 4 points.

Recruitment

For peer review only - http://bmjopen.bmj.com/site/about/guidelines.xhtml

As per WS1, children and their parents will be recruited via general, community and/or specialist paediatric services in English NHS Trusts, and children's nutrition and dietetic services attached to, or working into, these services. Based on estimated numbers of children using a home-blended diet we plan to recruit from around 20 NHS Trusts. For WS2 we will also plan to recruit from children's hospices in England and via social media, as recommended by the parent advisors for the study.

Families will be recruited primarily in routine clinic appointments with paediatricians or dietitians, but families may also be invited by post, telephone, and via social media. In WS2, families must be supported by a recruiting NHS Trust or children's hospice to take part, in order for clinical data to be obtained in the study. In WS2, clinicians will introduce the study and obtain consent from parents and children where possible for the study team to contact them. Consent to participate in the study will be sought by the study team, with appropriate consent / assent processes used depending on the age and capacity of participating children. For young adults (16-18yrs), an assessment of capacity in line with the Mental Capacity Act, will be undertaken by the clinician at the consent to contact stage.

All families will receive a full study information pack about the study, which will contain relevant participant information sheets and study consent forms depending on the child / young person's capacity. For young adults who lack capacity, we will identify an appropriate personal consultee to provide advice about the young adult's views and wishes about taking part in the study, using appropriate consultee information sheets and consultee declaration forms.

Consent processes are as follows:

- *Young adults age 16-18 years with capacity:* young adults and parents will provide separate consent for their own participation.
- *Young adults age 16-18 years without capacity:* no consent will be taken. Young adults will take part in the study if the consultee advises that they would not have any objections to taking part. Parents will consent separately for their own participation in the study.
- *Children and young people age 7-15 years who can understand information about the study and express an opinion about taking part:* parents will provide consent and the child / young person will provide written or verbal assent. A simplified version of the assent form will be used for children aged 7-11 years, with a standard version used for young people aged 12-15 years). Parents will provide separate consent for their own participation.
- *All other children and young people under the age of 16 years:* parents will consent for their child and themselves.

Data Collection

Data will be collected at three time points: at baseline and then at 9 and 18 months. At each time point, data on a range of outcomes as well as relevant clinical and feeding information will be collected from parents/children/young people and clinicians See Table 2 for summary of proposed outcomes and data sources (these are subject to change following WS1).

The majority of the data will be collected via parent questionnaire administered according to parent preference (postal vs on-line; parents will also be offered telephone interview). Parents whose children will also be required to provide dietary information via the online myfood 24 tool,[15] or via a paper food diary or telephone call with the research team if using the online tool is not possible. Where appropriate, participating children and young people will also be asked to complete a short

For peer review only - http://bmjopen.bmj.com/site/about/guidelines.xhtml

questionnaire (e.g. to self-report quality of life). Up to three reminders via text and/or post will be used at each time point. A small incentive voucher of £20 will be provided to each family after return of the questionnaires at each time point.

Clinical information (e.g. diagnoses, medications, anthropometry) will be collected from children's paediatrician and/or dietitian. Dietitians will also provide details about formula feeds used by participating children. Finally, we will use routine healthcare data through linkage undertaken by NHS Digital to the Hospital Episodes Statistics (HES) data (inpatient, A&E, outpatient) and the Office for National Statistics (ONS) death certificate data.

Table 2: Proposed variables and data sources for WS2

Variable	Type of Variable	Proposed Measure	Source	Timings of data collection (months)		
				0	9	18
**Participant characteristics / predictors**						
Age	Predictor	Date of birth	Parent	√		
Ethnicity		Census groups	Parent	√		
Deprivation		Index of Multiple Deprivation (based on postcode)	Parent	√	√	√
Parental educational attainment		Census groups	Parent	√		
Household composition		Number of children, marital / living status	Parent	√	√	√
Diagnosis			Paediatrician	√		
Co-morbidities			Paediatrician	√	√	√
All Medications			Paediatrician	√	√	√
Complexity		Disability Complexity Scale[16 17]	Paediatrician	√	√	√
Length of time gastrostomy fed at T0		Months/years	Parent	√		
**Comparator**						
Diet: Formula/blended	Main grouping variable		Parent	√	√	√

For peer review only - http://bmjopen.bmj.com/site/about/guidelines.xhtml

**Outcomes**						
Nutritional content of feeds	Outcome	Commercial for formula	Dietitian/Parent	√	√	√
		myfood24[15]	Parent	√	√	√
Anthropometric data	Outcome	Height or length<br>Weight<br>Triceps skinfold thickness or mid arm circumference	Dietician<br>Paediatrician	√	√	√
Gastrointestinal symptoms	Outcome	PedsQL Gastrointestinal[18]	Parent/ child	√	√	√
Child Quality of Life	Outcome	PedsQL generic module[19]	Parent/ child	√	√	√
Parental Quality of Life	Outcome	EQ5D5L[20]<br>Parenting Morale Index[21]	Parent	√	√	√
Healthcare use	Outcome	Client service receipt inventory[22]	Parent	√	√	√
	Outcome	No. hospital admissions<br>No. Accident and Emergency (A and E) attendances	HES data	√	√	√
Safety	Outcome	Tube blockages<br>No. of infections stoma<br>No. of gastrointestinal infections	Parent			
	Outcome	Number of hospital admissions and A and E attendance associated with child's gastrostomy/diet	HES & Parent	√	√	√
Family resource use	Outcome	Time preparing feeds; impact on other caring/parenting<br>Financial costs	Parent	√	√	√
Non-staff NHS resource use	Outcome	Cost of formula and packaging<br>Dietetic resources	Dietitian	√	√	√

Data Analyses

For peer review only

The data quality of each data item collected will be assessed when the data is collected or received by the research team. Appropriate attempts will be made to obtain missing or out of value data. A review of the collected data will be undertaken after the first 25 participants have completed baseline questionnaires to check for any systematic issues with the data collection.

A statistical analysis plan will be developed and signed off prior to analysis. Analysis will follow STROBE [23] and RECORD [24] guidelines. Descriptive statistics of clinical and demographic characteristics of the study population at baseline will be used to examine differences between the groups of children who are predominantly formula fed and those who use home-blended diets.

Children will be grouped into those who are on a predominantly blended diet or formula diet at baseline by:

- Home-blended group if most of their nutritional intake is provided via home blended diet. This categorisation will be informed by WS1 and in consultation with the SSC.
- Formula fed if most of their diet comes from formula.

Most of the proposed outcome measures will require scoring or aggregation before the statistical modelling can be undertaken:

- The Peds QL generic scale[19] and PedsQL Gastrointestinal symptoms module[18] will be scored as per the guidelines and transformed to a score of 0-100.
- The height (or length) and weight will be used to calculate an age and sex adjusted body mass index (bmi sds).
- The myfood24 data programme analyses the nutritional content the home-blended diet and will compute the calorie intake and the macro and micro nutrient content of the feed and any oral feeds. The same data for the formula fed group will have been obtained, via the dietitian, from the commercial supplier.
- The parent reported number of site infections and other tube related complications will reported as total counts for each child.
- The diagnostic (ICD10[25]) and procedural codes (OPCS) in the HES data will be used to identify admissions which were related to complications of the gastrostomy tubes or infections. The number of admissions and A & E attendances will be calculated for each child. Length of stay for each admission will also be calculated for the resource use analyses.
- Parent quality of life: the EQ5D VAS is scored 0-100 and the 5 component scale of the EQ5D-5L will be converted to a single score using a UK specific value set [26] . The 10 item Parenting Morale Index is scored from 0-100.

For all outcomes we will report the baseline score, follow-up scores and change score.

Assessing safety (objective 2) and benefits (objective 3):

The safety and benefits of blended diet compared to formula diet will be assessed using multivariable regression analyses. The type of regression will depend on the outcome of interest; logistic (tube blockage, appropriate nutritional content; yes/no), linear (PEDSQL gastrointestinal module score, BMI sds or upper arm circumference, calories, PMI, EQ5D), Poisson or negative binomial (number of A & E or hospital admissions for infections or complications of gastrostomy tube). Each analysis will account for the multiple confounding factors in this population (age, underlying diagnoses, comorbidities, outpatient attendance, parental factors, socio-economic status) and the main covariate of interest will

For peer review only - http://bmjopen.bmj.com/site/about/guidelines.xhtml

be feeding status (blended vs formula). Study site will be added as a random effect to the models to allow for site level variation. Estimates and 95% CIs will be reported from the regression model for each outcome measure.

The flow of participants through the study will be detailed including the number of individuals contributing to each analysis. The amount of missing data will be summarised for each outcome measure and multiple imputation will be used to assess the robustness of the results. Results will be compared to the complete case analyses and important differences discussed. Sensitivity analyses will be considered to explore departures from the MAR assumption.

Measurement of cost and outcomes (objectives 3 and 4)

There is a lack of robust evidence around the cost-effectiveness of alternative feeds for gastrostomy fed children. To address this, we will describe the costs and outcomes for those children with a formula diet and with a –blended diet(addressing objectives 3 and 4). The formula feed group will act as the treatment as usual.

Generating cost estimates

Unit costs for healthcare interactions will be collected from published sources (for example, PSSRU Unit Costs of Health and Social Care) and applied to the relevant resource use. The costs of healthcare interactions will be calculated by the product of unit cost and resource use analyses.

The cost of non-healthcare interactions, including the cost of the blended diet constituents and time taken to prepare will be estimated separately using published estimates where feasible. Sensitivity analyses will be conducted where alternative assumptions would generate substantially different cost estimates (for example, if there are substantial differences between parental report and HES data).

Generating estimates of outcomes

We will describe and summarise estimates of parent changes in HRQoL (EQ5D-5L) and child (either PEDS QL or QoL questionnaire [27]). We will describe these for both groups within the cohort.

We will report total costs, mortality and adverse event rates associated with both home-blended and formula feeds in a cost-consequences framework.

Long term follow up (objective 5)

The utility of routine data sources as an option for long term follow up of study participants will be assessed by examining the concordance between parent reported data on A and E visits and hospital admissions due to infections or complications of their gastrostomy tube with HES data for the corresponding time period. Concordance will be assessed using the kappa statistic both for the total sample and separately by the home-blended and formula fed groups.

If there is concordance between the parental reported infection and gastrostomy related healthcare usage and HES data, long term follow-up would be possible by obtaining further extracts of HES data and ONS death certificate data. The HES data will provide future information on admissions and A&E visits due to infections and complications of the gastrostomy (blockages, revisions, replacements). The ONS data will provide date and cause(s) of death if the child has died.

Patient and Public Involvement

For peer review only - http://bmjopen.bmj.com/site/about/guidelines.xhtml

Six parents whose children were gastrostomy fed were involved in the development of the study design and methods, and plans for public involvement. In particular, they helped to identify outcomes to propose for WS2, develop appropriate recruitment methods for both workstreams (e.g. recommended that we use social media), and they chose the study title 'YourTube'.

During the study, our primary mechanism for public involvement will be via a Project Advisory Panel comprising 4-5 parents and 2-3 young people with gastrostomy experience. The panel will meet twice per year at key points in the study, and be involved at other times when needed via telephone, email or in person depending on the task. Panel members will be supported by the study team, and receive training for specific tasks when needed.

Members of the panel will be involved in the following:

Study oversight: A minimum of two members of the panel will also be members of the SSC.

Developing study materials: reviewing participant information sheets, consent forms and interview schedules; piloting of WS1 topic guides and materials; development of guidance for parents taking part in WS2; piloting of WS2 questionnaires and data collection tools.

WS2 study design: selection of outcomes to be included in WS2, and input on the acceptability and feasibility of proposed measurement tools.

Study interpretation: input on the meaning of study findings, and development of key messages for policy and practice

Study dissemination: help to produce information resources for parents, children and young people at the end of the study; identify routes for dissemination and assist in the dissemination of the study outputs directly via their own networks.

Ethics and dissemination

Approvals from the English National Health Service Research Ethics Committee and Health Research Authority have been obtained for this study (REF 19/YH/0028).

The study findings will be disseminated in a final report to the funder, academic publications and resources for professionals and parents, children and young people.

References

1. Together for Short Lives. A Guide to Children's Palliative Care. 4th ed. Bristol, 2018.
2. Smith TM, Hirst A, Stratton R. Artificial Nutrition Support in the UK 2000-2010. Annual BANS Report: BANS 2011.
3. British Dietetic Association. Practice Toolkit; Liquidised Food via Gastrostomy Tube 2017 [Available from: https://www.bda.uk.com/professional/practice/liquidisedtoolkit accessed 1/9/2017 2017.
4. Armstrong J, Buchanan E, Duncan H, et al. Dietitians' perceptions and experience of blenderised feeds for paediatric tube-feeding. *Archives of Disease in Childhood* 2017;102(2):152-+. doi: 10.1136/archdischild-2016-310971
5. Johnson TW, Spurlock A, Pierce L. Survey Study Assessing Attitudes and Experiences of Pediatric Registered Dietitians Regarding Blended Food by Gastrostomy Tube Feeding. *Nutrition in Clinical Practice* 2015;30(3):402-05. doi: 10.1177/0884533614564996
6. Breaks A, Smith C, Bloch S, et al. Blended diets for gastrostomy fed children and young people: a scoping review. *Journal of Human Nutrition and Dietetics* 2018;31(5):634-46. doi: 10.1111/jhn.12563

For peer review only - http://bmjopen.bmj.com/site/about/guidelines.xhtml

7. Brown S. Blended food for enteral feeding via a gastrostomy. *Nursing Children and Young People* 2014;26(9):16-20.
8. Siden H, Tucker T, Derman S, et al. Pediatric Enteral Feeding Intolerance: A new prognosticator for children with life-limiting illness? *Journal of Palliative Care* 2009;25(3):213-17.
9. Romano C, van Wynckel M, Hulst J, et al. European Society for Paediatric Gastroenterology, Hepatology and Nutrition Guidelines for the Evaluation and Treatment of Gastrointestinal and Nutritional Complications in Children With Neurological Impairment. *Journal of Pediatric Gastroenterology and Nutrition* 2017;65(2):242-64. doi: 10.1097/mpg.0000000000001646
10. Creswell JW, Plano Clark V, Hanson W. Handbook of mixed methods in social and behavioral research. *Advanced Mixed Methods Reserach Designs* 2003;212
11. University of Stirling. Talking Mats 2018 [Available from: https://www.talkingmats.com/ accessed 15/3/2018 2018.
12. Rabiee P, Sloper P, Beresford B. Doing research with children and young people who do not use speech for communication. *Children & Society* 2005;19(5):385-96. doi: 10.1002/chi.841
13. Miles M, Huberman M, J S. Qualitative Data Analysis. London: SAGE 2013.
14. Ritchie J, J L. Qualitative Research Practice. London SAGE 2003.
15. Carter MC, Hancock N, Albar SA, et al. Development of a New Branded UK Food Composition Database for an Online Dietary Assessment Tool. *Nutrients* 2016;8(8) doi: 10.3390/nu8080480
16. Horridge KA, Harvey C, McGarry K, et al. Quantifying multifaceted needs captured at the point of care. Development of a Disabilities Terminology Set and Disabilities Complexity Scale. *Developmental Medicine and Child Neurology* 2016;58(6):570-80. doi: 10.1111/dmcn.13102
17. Horridge KA, McGarry K, Williams J, et al. Prospective pilots of routine data capture by paediatricians in clinics and validation of the Disabilities Complexity Scale. *Developmental Medicine and Child Neurology* 2016;58(6):581-88. doi: 10.1111/dmcn.13101
18. Varni JW, Bendo CB, Shulman RJ, et al. Interpretability of the PedsQL (TM) Gastrointestinal Symptoms Scales and Gastrointestinal Worry Scales in Pediatric Patients With Functional and Organic Gastrointestinal Diseases. *Journal of Pediatric Psychology* 2015;40(6):591-601. doi: 10.1093/jpepsy/jsv005
19. varni JW. The PedsQL measurement model for the pediatric quality of life inventory 2017 [Available from: http://www.pedsql.org/.
20. Herdman M, Gudex C, Lloyd A, et al. Development and preliminary testing of the new five-level version of EQ-5D (EQ-5D-5L). *Quality of Life Research* 2011;20(10):1727-36. doi: 10.1007/s11136-011-9903-x
21. Benzies KM, Trute B, Worthington C, et al. Assessing Psychological Well-Being in Mothers of Children with Disability: Evaluation of the Parenting Morale Index and Family Impact of Childhood Disability Scale. *Journal of Pediatric Psychology* 2011;36(5):506-16. doi: 10.1093/jpepsy/jsq081
22. Chisholm D, Knapp MRJ, Knudsen HC, et al. Client Socio-Demographic and Service Receipt Inventory - European Version: Development of an instrument for international research - EPSILON Study 5. *British Journal of Psychiatry* 2000;177:S28-S33. doi: 10.1192/bjp.177.39.s28
23. von Elm E, Altman DG, Egger M, et al. The Strengthening the Reporting of Observational Studies in Epidemiology (STROBE) Statement: Guidelines for Reporting Observational Studies. *PLOS Medicine* 2007;4(10):e296. doi: 10.1371/journal.pmed.0040296
24. Benchimol EI, Smeeth L, Guttmann A, et al. The REporting of studies Conducted using Observational Routinely-collected health Data (RECORD) Statement. *PLOS Medicine* 2015;12(10):e1001885. doi: 10.1371/journal.pmed.1001885
25. World Health Organisation. International Statistical Classification of Diseases and Related Health Problems. 10 ed. Geneva, Switzerland: World Health Organisation 1992.
26. Devlin NJ, Shah KK, Feng Y, et al. Valuing health-related quality of life: An EQ-5D-5L value set for England. *Health Economics* 2018;27(1):7-22. doi: 10.1002/hec.3564
27. Zaidi T, Sudall C, Kauffmann L, et al. Physical outcome and quality of life after total esophagogastric dissociation in children with severe neurodisability and gastroesophageal reflux, from the caregiver's perspective. *Journal of Pediatric Surgery* 2010;45(9):1772-76. doi: 10.1016/j.jpedsurg.2010.04.022

Author's contributions

For peer review only - http://bmjopen.bmj.com/site/about/guidelines.xhtml

MO and LF drafted the first version of the manuscript. LF,BB,CH,JT,GR,KH,JC contributed to the conception of this study. LF, BB, CH,GR,KH, JC, JM and AM contributed to the design of the study. All authors reviewed the manuscript prior to submission.

Funding statement

This study is funded by the National Institute for Health Research Health Technology Assessment programme (ref 17/76/06). This was a commissioned call so the funder identified the topic area for this study but will have no role in the study design, data analyses or conclusions in this study.

Competing interests statement

None declared

For peer review only

For peer review only - http://bmjopen.bmj.com/site/about/guidelines.xhtml
