# Supplementary material for: ‘Your Tube’: the role of different diets in children who are gastrostomy fed: protocol for a mixed methods exploratory sequential study
Source: BMJ Open. 2019 Oct 9;9(10):e033831. doi: 10.1136/bmjopen-2019-033831 (PMC6797291; doi:10.1136/bmjopen-2019-033831)
Supplement: Reviewer comments [file bmjopen-2019-033831.reviewer_comments.pdf]

PEER REVIEW HISTORY

BMJ Open publishes all reviews undertaken for accepted manuscripts. Reviewers are asked to complete a checklist review form (**http://bmjopen.bmj.com/site/about/resources/checklist.pdf**) and are provided with free text boxes to elaborate on their assessment. These free text comments are reproduced below.

ARTICLE DETAILS

**TITLE (PROVISIONAL)**	'YourTube' The Role of Different Diets in Children who are Gastrostomy Fed; protocol for a mixed methods exploratory sequential study
**AUTHORS**	Taylor, Johanna; O'Neill, Mark; Maddison, Jane; Richardson, Gerry; Hewitt, Catherine; Horridge, Karen; Cade, Janet; McCarter, Alison; Beresford, Bryony; Fraser, Lorna Katharine

This article was not externally reviewed at BMJ Open. Protocols that have been independently assessed prior to submission to BMJ Open are usually fast-tracked to publication on the grounds that further substantial changes will not be possible. This independent assessment will usually be external, independent review for both a substantial grant award from a non-commercial or government funder and ethics approval.
